# Supplementary material for: A SNP variation in an expansin (EgExp4) gene affects height in oil palm
Source: PeerJ. 2022 Mar 16;10:e13046. doi: 10.7717/peerj.13046 (PMC8934041; doi:10.7717/peerj.13046)
Supplement: Supplemental Information 1 [file peerj-10-13046-s001.pdf]

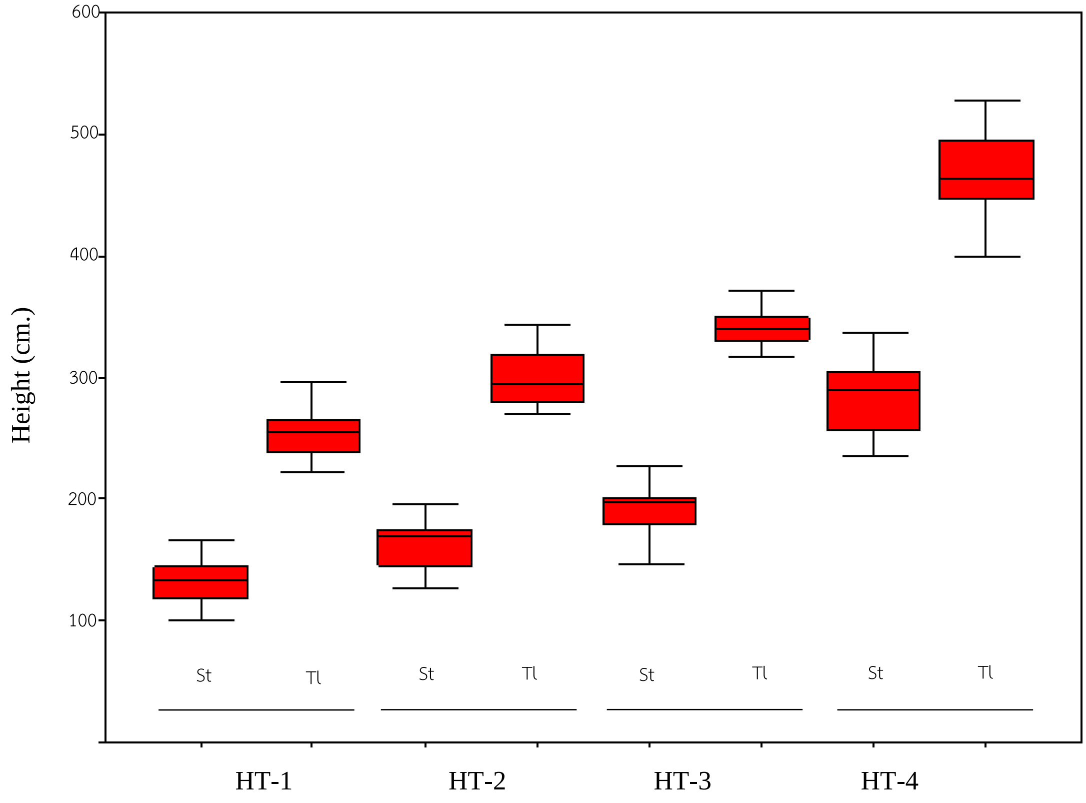

Fig. S1 Comparison of height distribution in short (St) and tall (Tt) oil palm groups from height data, including HT-1, HT-2, HT-3 and HT-4, of the GT population. cm = centimeters
