# Supplementary material for: A SNP variation in an expansin (EgExp4) gene affects height in oil palm
Source: PeerJ. 2022 Mar 16;10:e13046. doi: 10.7717/peerj.13046 (PMC8934041; doi:10.7717/peerj.13046)
Supplement: Supplemental Information 2 [file peerj-10-13046-s002.pdf]

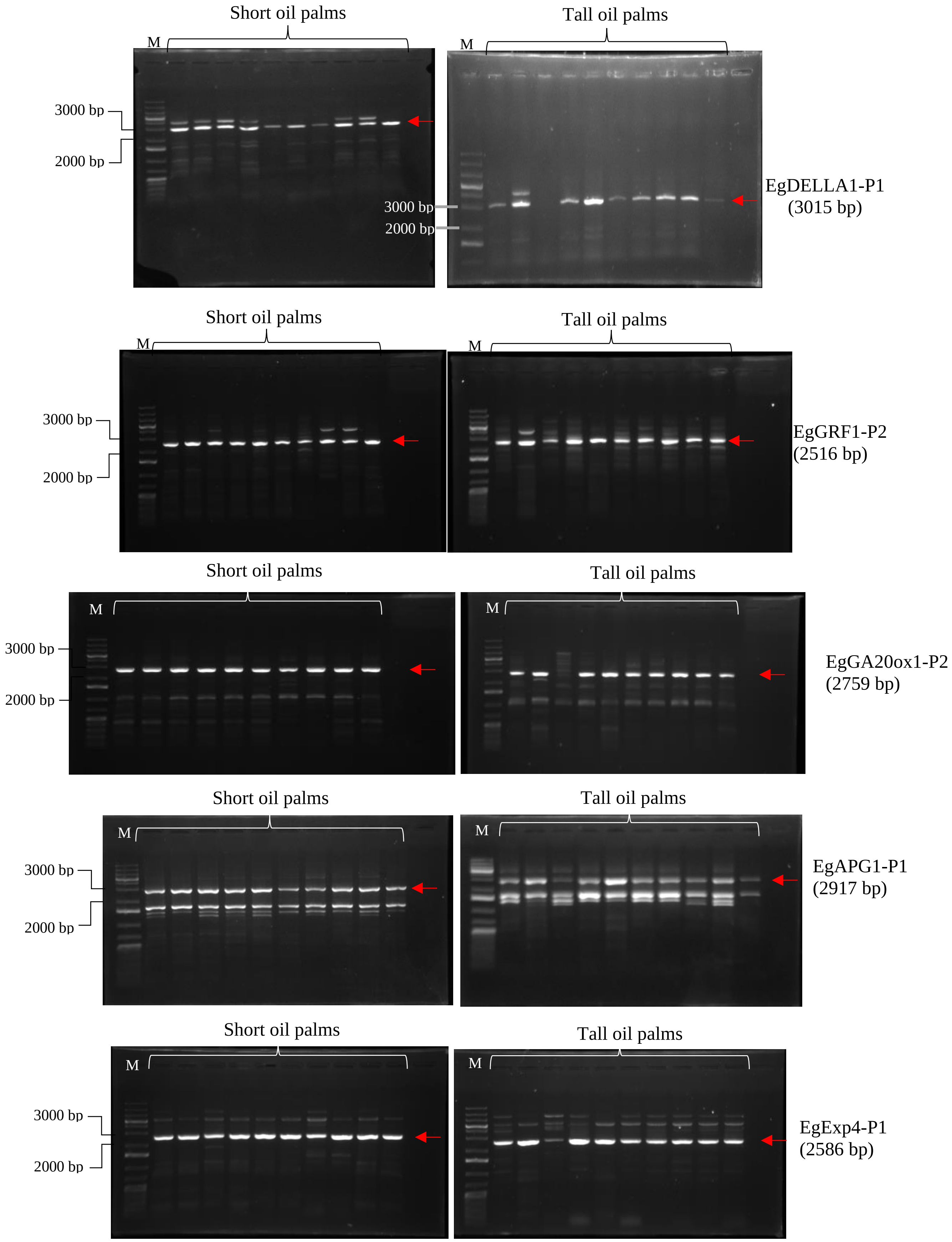

Fig. S2 Example of PCR products amplified from the height-related genes by the gene-specific primers, including EgDELLA1-P1, EgGRF1-P2, EgGA20ox1-P2, EgAPG1-P1 and EgExp4-P1 with bands close to the expected sizes from 2516-3015 bp (shown by red arrows). M = 1Kb Plus DNA ladder
