# Supplementary material for: A SNP variation in an expansin (EgExp4) gene affects height in oil palm
Source: PeerJ. 2022 Mar 16;10:e13046. doi: 10.7717/peerj.13046 (PMC8934041; doi:10.7717/peerj.13046)
Supplement: Supplemental Information 3 [file peerj-10-13046-s003.pdf]

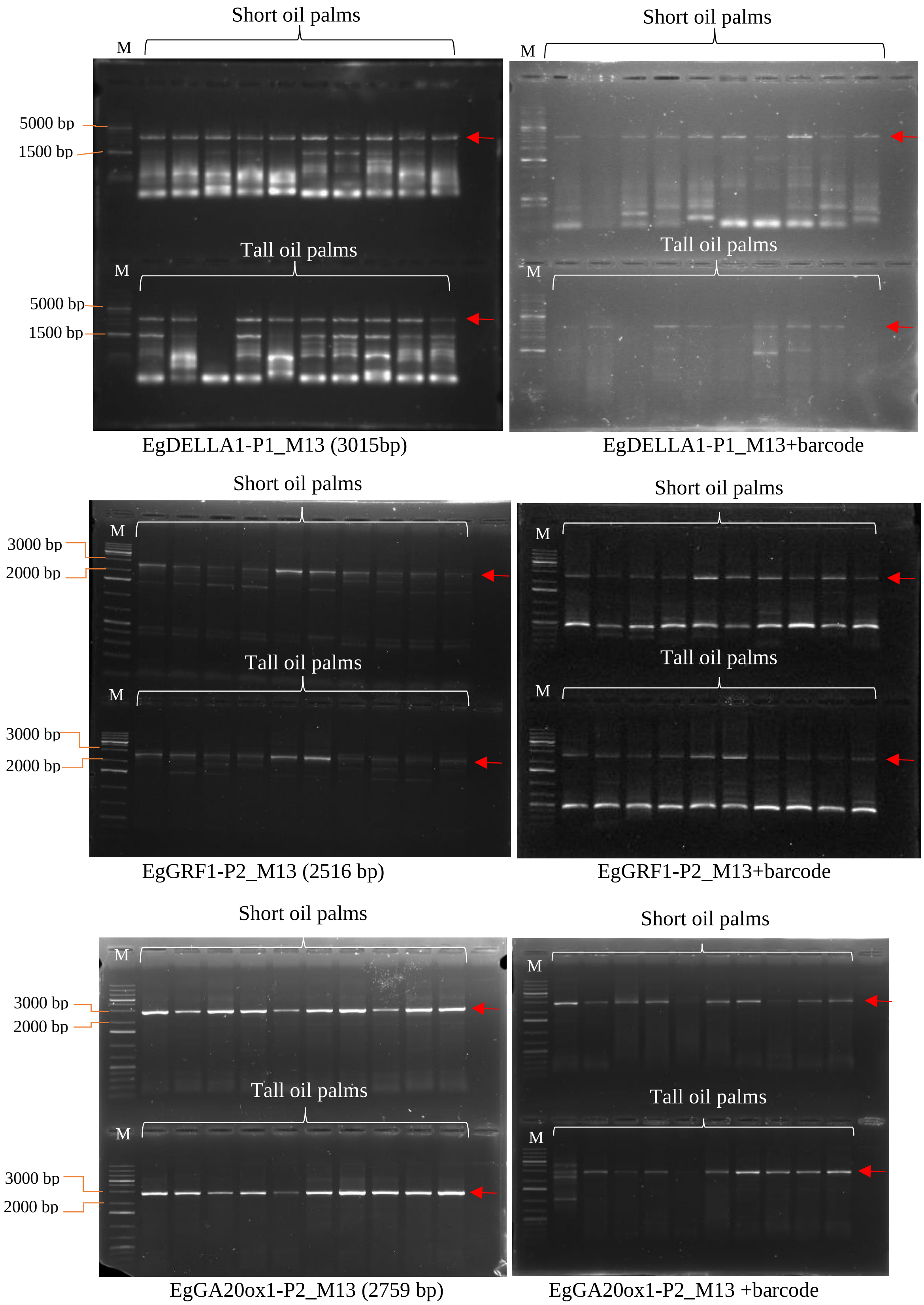


**Fig. S3** (continuted)

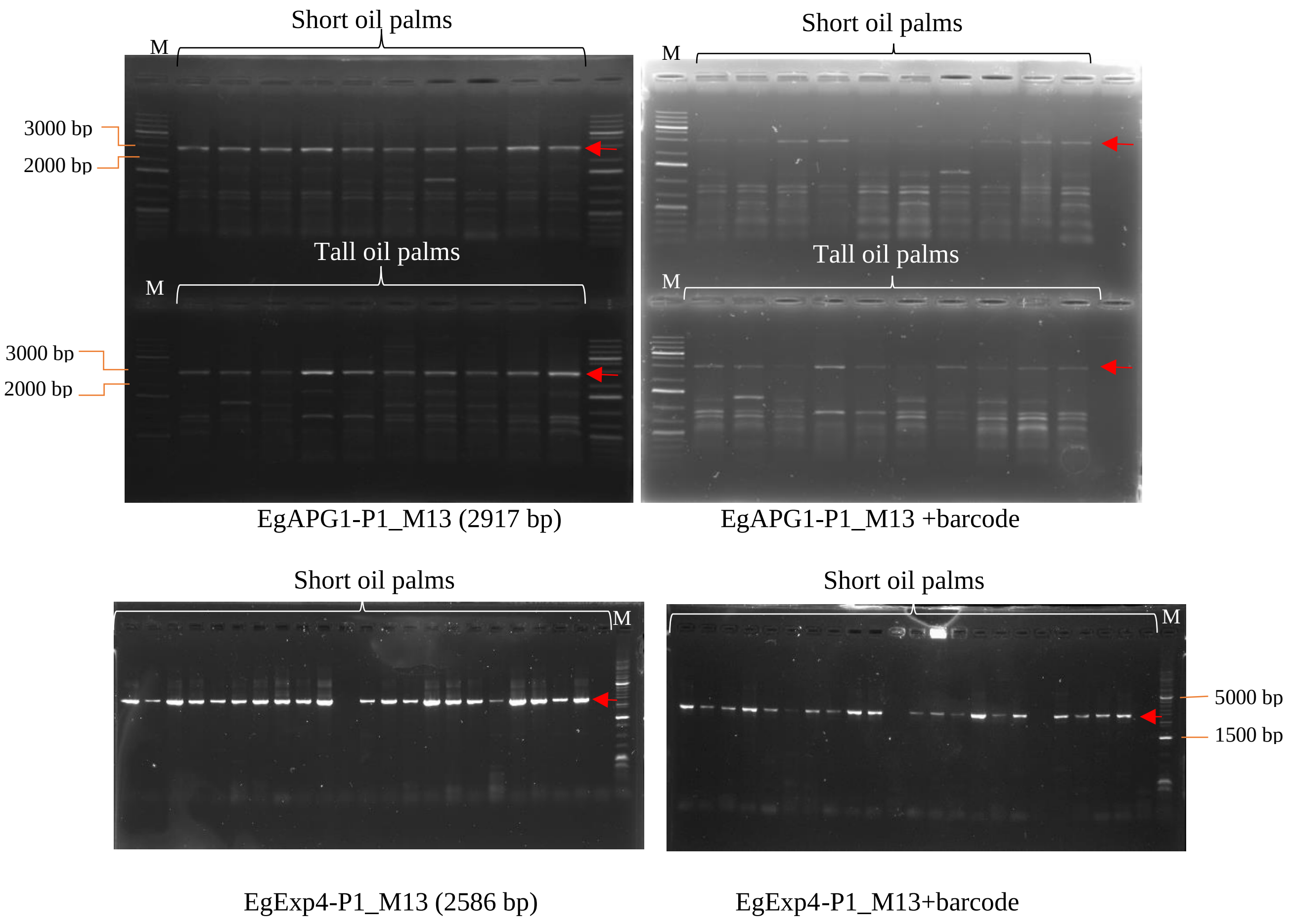


**Fig. S3** Example of PCR products amplified from *EgDELLA1*, *EgGRF1*, *EgGA20ox1*, *EgAPG1* and *EgExp4* with bands close to the expected sizes from 2516-3015 bp (shown by red arrows) by the M13-tagged primers (Gel images on the left side) and the barcode-tagged M13 primers (Gel images on the right side). M = 1Kb Plus DNA ladder. The high-intensity band in the sample loading well on the last gel was incompletely dissolved SYBR® Safe DNA Gel Stain
