# Supplementary material for: A SNP variation in an expansin (EgExp4) gene affects height in oil palm
Source: PeerJ. 2022 Mar 16;10:e13046. doi: 10.7717/peerj.13046 (PMC8934041; doi:10.7717/peerj.13046)
Supplement: Supplemental Information 4 [file peerj-10-13046-s004.pdf]

***EgDELLA1* Full length information**

Length: 3015 bp (Same as report)
START Codon: 923-925 (Checked)
STOP Codon: 2659-2661(Checked)
SNP T → A: 2100
SNP G → A: 2248

BLAST Information
Name: DELLA protein RGL1-like [*Elaeis guineensis*]
Sequence ID: XP_010938288.1
Exon 1: 923-1375
Exon 2: 1495-2792

= Primer
= START/STOP codon
= Insertion T → TA. Insertion found in some short oil palm
= Nucleotide change (SNP) T → A. Base A found in some short oil plant
= Nucleotide change (SNP) G → A. Base A found in some short oil plant
Underline = Exon

EgDELLA1 Full Sequence

```
1     TTTTCGtACA TTCGGCTCTG TGCAATGCAT GAATCTTTGA TTAATTTTCT

51    TATAATTCCT TACACATGTG ATTGGGCTCT CCAAGATTCT TGGTTGCTTC

101   CTGTCTTATT GTCAGCTTTT GAAAGCAACT TCTTGCTATG GTCTATAGCA

151   ATGGGTAGGT CTTTAATGAA ATAGAGAGAA ACTGGTCCGG AATTTGGAAT

201   TTGTGGGTAC CAGTTCTTTG CTTGTCAATT GCTCCTATTA CATATCTTGG

251   AAGAATTTTC GGTCCAGACA AATAGCCGGT GCCATTTGAT ACCTTGTTGC

301   AATGAAACCA TTAAAAgACT GCATTCTTAA TTAAATATAA TGAAGTTTCT

351   AATCTGAAaG ATTTTATTAT TGAGGTTTCT TGATATGTGG GCCCTCTGCA

401   ACTAACTCGT TGTTGGGTCA TCGTGCACAC CGTAATTGGT AACACCGTCC

451   AATCTgCTCA CACCTTCTGC TTCGATTTGT ATTTGGTTTA ATACAATTTG

501   GTCCTCTCAC TCCATGATTA GTTGCCATGG AGCCAGCTAC TCACCAACTG

551   TTTGCATccA GAACCACATA AATGAAATGA CcGcAAAGTA TAAAAATTGA

601   CGCCGCTATT GATCAATGAT TAATACTATG TGACTTGTTC TTAGCTAGCG

651   AAAGCCAaTG GAAAACACTT AAAACAAttt gCTAATACCA TGGCTTGGTA

701   GGACTGTCTT ATGCTAACAA TGTTGTCATG GATTACTTCA GCTTTCCTGT
```

[cont. *EgDELLA1* Full Sequence]

751 CATTTCCCCT TGTTGGATTC CCTTCACTTT GTGGATTTCT AGCACCAAAG

801 TTTCCAACAT TTTGGATCAC TCAGAACTCT TCAACTCATA AACATTTATC

851 ATAGCCCATC AAAATCTGGA ATTGTCAAAT CTTGAGAAGA AGTTTAGCAC

901 TACACAGAGG TCTGCTGCGA AGATGCTGAG CTCTCCCTTC TCTTTtCCGT

951 GTTTCGATTG TGATGGTACA CAGCAAGGCT ACACCCCTCT GCAGGGATTT

1001 GAAGAAGAAG AGTGCATTGA TGACCTTTGG TCTGGTTACA ATTCATACCA

1051 TGATGGCCCT TTTGAGAAGG TAACCCCCTT GCTATCCAGT GAACAGCAAT

1101 GCTTCCAAGA TCTTGCAGTG ATGGATGACA TGCAGCTTGA TACAATTTCT

1151 GCAGCAATCC AGTCCTTGGA CAAACCTTTG GCCCAGGAGA CACAGATACA

1201 GCAGCAGCCA GTGGTGCCTG AAATCCCTGA GCTTCTTGGA TCAGAGGAGA

1251 AGGCATCCCC AGTGAACTTC TCATCATTAG AGCTTCTGAA CAACTACAGA

1301 TCAAGCTACA GTCGCCTGAA CGGAGAAAAG CTGAATGAAC CGATTGACGG

1351 GGTCACGCAG CCAGTGGAAG GAGGCAAtGA GTTGTCGACG GAGGAAATCA

1401 TGAGAATTGC TGCTGTCCAT TTTGTTCAGG TGTCAACCCA TAAGAAAGT

1451 GATCCTTTCG TGCCTTGGCA TCCTGCTAAC TTCTTATTTT AGGCCTCACA

1501 AATGAGGAAA GAGAGAACAT CGGCCTCGCA AACCTTCTtC TGGCTGCTGC

1551 GGAGAAGATA AGCAACCAGC AATATGATCG AGCTAGCAAT TTACTTCAGG

1601 AGTGTCACAA ACGGTCATCC AAAACCGGAA ATCCAGTTCA GAGAGCTGTT

1651 TTCTACTACA CAGATGCATT ACAAGAAAGG ATCGATAGAG AAATTGGAGG

1701 GCTTTCATCG AAGGTTCCAA AGGATGGGGG CATGACGGCA CCAGAGCTCA

1751 TCAAAGCATT GTTATCTGAC CACCCATTGC ATGTGGCACT CTACAATAAG

1801 CTGCCATTCT CACAAATCGA TCAATTCACT TCGGTGCAGA CCATGTTGGA

1851 CAACATGGCC ACCGCAaGGA AAATCCATCT GATCGATCTC TCGATCCAGC

1901 ATGGGATCCA GTGGGCTGTT TtCTTGCAAG CACTGGCCAC AAGAACAACA

1951 TGTCCAATCG ACCGTCTGAA GATTAGTGCA ATAGATACAT CAGAAGAGGC

[cont. *EgDELLA1* Full Sequence]

2001 AATCACTGCA ACTGGTAACA GGCTGGTAAG CTTTGCGGAA ACTTTAGGCT

2051 TGCCCTTAAG TTTTAAAGCA GTGGCAGTCT CAGATCTCAA GGATCTCAAT

2101 GAAGAGATGT TTGAATTGGA GGAGGGCGAA GTGATCGGTG TCCGCTCTTC

2151 GATGTTTATG AGCAGCTTGA TTGTCAGGCC TGACATACTT GAGCATGTCA

2201 TGAGAGTAAT GCGCAAGATT AAGCCATACG TAATGATAGT TGCAGATGTT

2251 GAAGCTAAAC TTAATTCCCC ATCTTTCATC AACCGCTTCA CTGAAGCCTT

2301 GTTTTTCCAC AGCGCCTTAT TTGACTTCCT GGAAGGCTTT CTGAAGAGGG

2351 ATGATGAAAG TAGAATGCTT GCAGAGGGAT CCTTCATTTC ACAGGGAATA

2401 CGAAACATCA TAGCGACCGA GGGCCATGAG AGGCTGGCCA GGTATGTAGG

2451 CATCAGCGTG TGGAGGTCAT TCTTTGCTCG TTACGGCTTC ATCGAGACTG

2501 AGATGAATGA GTGGTCACGT TACCAAGCTA GACTGCTAGT TAAGCAGTTT

2551 GCTAATGGCC ACTTCTGCAC ACTTGACAAG AATGGGAAGG CCTGGACCAT

2601 GGGGTGGAAG GGGACCCCCT TGCTTTTTAT CTCTGCTTGG AAGTTTCAGC

2651 AGGCAGGATA GGAAGAATTT TTCTAGTTCT CAATCTGATA TTTATATCAT

2701 AGCACTAGGT TTTATTATGC CATATAGGAG CAGAAAGCAA GTATGAGGTG

2751 TTGGTCCTGA AATGTTGCCA AAAAGTTTAT AACTTGCTCT TAGAATTCTT

2801 GTAGTCCTTT TTATGCCATG TAACCTCTGG AAGCTGAAGT ATTGAGTATA

2851 TACTAGCATG TCACGTTCCC TCCTCTTGGT GGATCTTCGA GTCTTGTGCT

2901 CAGGTCTCAC ATTATGCAGT TATGTATGAT ACACAGCATG AATTTTTTTC

2951 CTGAAAGTAA ATAACCAATA TTATTCCTAT AAGCTAACTC GCATCTTCTC

3001 GCTTTCGAAC AGGAT

///End of sequence

Fig. S4 The insertion and SNP variations are illustrated on the *EgDELLA1* reference gene sequence, which has a full-length of 3015 bp (oil palm draft sequences of Malaysian Palm Oil Board (MPOB), (http://genomsawit.mpob.gov.my/genomsawit/)).
