# Supplementary material for: A SNP variation in an expansin (EgExp4) gene affects height in oil palm
Source: PeerJ. 2022 Mar 16;10:e13046. doi: 10.7717/peerj.13046 (PMC8934041; doi:10.7717/peerj.13046)
Supplement: Supplemental Information 5 [file peerj-10-13046-s005.pdf]

***EgGRF1* Full length information**

Length: 2516 bp (Same as report)
START Codon: 772-774 (Checked; Different with report)
STOP Codon: 2112-2114 (Predicted)
Deletion TATA → T   : 1044
Insertion G → GT   : 1100
Insertion A → ATCTC   : 1553
Deletion ATCTCTC → A : 1553

BLAST Information
Name: growth-regulating factor 1 [*Elaeis guineensis*]
Sequence ID: XP_010932423.2
Exon 1:   602-973
Exon 2:   1146-1544
Exon 3:   1657-2091
Exon 4:   2196-2504 [outside this sequence]

= Primer
= START/STOP codon
= Deletion TATA → T. Deletion found in some short oil palm
= Insertion G → GT. Insertion found in some short oil palm
= Insertion A → ATCTC. Insertion found in some short oil palm
= Deletion ATCTCTC → A. Deletion found in some tall oil palm
Underline = Exon

```
1     TCATTGTTCC ACACTTCCAC ATCTTTACCA TTACTTTGTG TTTTtGTTTA

51    AACCCCAAAC AACTCTTTtG TTTATGAGTT TCCGTAAAGC TGGCAGGAGA

101   CAACAGCAGC AAAAGGCACC AGAGCACAGC AGCTTGCTGC AGAGATAGGA

151   AGAGATAGAG AGAGAAATAT AGAGCTAAAG ATAGAGAGAG AGAGCGAGAG

201   CCCTGCTCTC CTCTGGTTGG CAGACCTGCT CCTGCCCCTC CTGCTGCTGC

251   TCCTGCTCTC AGCCATAATA ATAAGAGCAG AGTCTACTGC TCCCAGGTGG

301   CAGAACCACC TGCAACCATT CTGACAGCCA TTAGCATTAA ACTGGTGGCT

351   TTTACCCGAG TGACATGGAA AGGCATGCAC CATATATCAT CACTCCACCA

401   CCCAACCCTT TTGCTTGCCC TTTTTCTTTt CTTTTGTTAT ATATTCTATA

451   AGCCTCAGGA GTCTTGATTG ATCCATGCAA CTACACAGAA GATTCTGCAG

501   CCTACACTCT GCCTCTTGAC TGAATGACCC ATCCTATGCA CCCACATCAT

551   AAAaTAATCC CCATCCTAAT ATTATTGTTT ATTATAATTT CTGAAACTCC
```

[cont. *EgGRF1* Full Length Sequence]

601 TCAGCGTTAA ATGCAGGCAG CCCCCCcTTT CCTCTCTCCT ATTTGTAGCA

651 TCTAACTCCA TACAAATATA TCAATATATG TAAGCAGGTG TAGCTTGTCT

701 CCTCTCTCAT TTCCTCTCTG TTTTCACCTC TTCTCCTCTT GAAGTTGATA

751 GGTTTGTTCT TTGGCTTTGA GATGATGATG AGTGGGAGGA ACACCTCTAG

801 GTATCCCTTC ACACCCTCTC AGTGGCAGGA GCTGGAGCAC CAGGCTCTCA

851 TCTTCAAGTA TATGGCCTCA GGAATCCCCA TCCCCTCTGA TCTCATCCTC

901 CCCATAAGGA GGAGTTTCCT GCTGGATTCC ACCATCCCCT CCCcAACTTT

951 GGCCTTCCCT CCCCAGCCTT CAAGTAAGTT TCTTCTCTCT CTTtCTCTCT

1001 CTCTCTCTCT ATATATATAT atgTGTGTGT GTGTGTaTAT ATATATAaTA

1051 GATAGATAGA TAGATAGATA GATATTTGAA TTTACTTCTC TTCCATGtGG

1101 TTTTTtGAGa GGAAAAatGT TTTGATCTGG GTCTCTGTAT GGCAGTGAGC

1151 TGGGGCTGGA ACCAGAtGGG TTTTGGCAGG AAGGCAGAGG ATCCAGAGCC

1201 AGGGAGGTGC AGGAGAACTG ATGGGAAGAA GTGGAGGTGT TCCAAAGAGG

1251 CCTACCCAGA CTCCAAATAC TGTGAGAGGC ACATGCACAG AGGCAAGAAC

1301 CGTTCAAGAA AGCCTGTGGA AATGTCTTTG GCCACCAGCC TCCCCTCTTC

1351 CACCTcTTCT TCCACCCCTT CCACCTCCCA CACCCCCTCC CTCCACCCAC

1401 CTCCTCTCTC CATCTCCACT CCAGACTCTC ACCAGCTTCT GTATCCCTCC

1451 CACCCCTCTT CTAGGATACC TGGTGTTGGC CTCAACACTC CCCACTTCAA

1501 TCTACATGCT CCATCCTACA CCCAAGCTGA CAAGAACTAC AGGTTTGGAT

1551 TCATCTCTCT CTCTCTCTCT CTCTCTCTCT CTCTCTCTCT CTCTATCTAT

1601 CTATCTATCT ATCTATCTAT CTATAACTAG TGGTATGAAT GACTGGAATG

1651 GCAGGTATTG TCATGGAATG AAGGATGTGG ATGAGTACTG CTTCTTTTCA

1701 GAGGCCTCAG GAAGTGAGAA GGAGAGCTCA TGGCAGTTCA GGCCACTAGG

1751 GATGAGTTCA CTAGGAGATA CCAAACAGGG GAGTTCTGCT CTGCAGCAGA

1801 GCACCTACTC TCACTGTGGA AGGGATGCAG AGGAGAAGCA GCAGCACTGC

[cont. *EgGRF1* Full Length Sequence]

1851 TTTGTTCTGG GGGCTGATTT CAAGCTGGAG AGGCCTGTGA AGGTGGAGAG

1901 GGAGCAGGAG GAGGGCCAGA GGCCTCTGCG CCACTTCTTC GATGAGTGGC

1951 CgCAGAGGAG CAGAGACTCA TGGGTGGATT TGGAACAGGA GAACTCCAAC

2001 CAGGCATCTA CCTACTCCAA GACCCAACTC TCAATCTCCA TCCCCATGTC

2051 TCATCACGAC TTCCCGGTCA CCGGTTCCCG ATATCGCAAT GGTGTAGTCC

2101 CCACTGCCCA ATGATCCTTC CATCCCAATA TATTGTCTTC TCCATTGTTG

2151 TATTAATCCT TTTATCACCC ATCTTTCAAT TTAATTGTGT TGCAGATGAC

2201 TGAGTTCTGA AAGGCTTTCT GTGCGAGCTG GGACCAAAGA ACTCATCAAA

2251 GCTCCATGGT ATCTTGTCAG ACAGAAGCTG TTTTCCAATG GGCTTCAATG

2301 CCCTCCTTGT GTATTACCCA TTGTTTGTTC TTTTTCTTTG TTGACTGTGT

2351 CAGGAAGAAC TTTCACATTC CTCATTTTGT TtCCTCTGGC TTTGTCTGTT

2401 CCAATGGCTG AGGAGAAGTC TTTAGGAATG AGGAGATCGT TTCGTCTATA

2451 ATtGTCTATC ATGTCATCAT CTATTATCTC TATCATCTAT CTGTAACCAG

2501 TCTTCTGATC TTCTCC

///End of sequence

Fig. S5 The insertion and deletion variations are illustrated on the *EgGRF1* reference gene sequence, which has a full-length of 2516 bp (oil palm draft sequences of Malaysian Palm Oil Board (MPOB), (http://genomsawit.mpob.gov.my/genomsawit/)).
