# Supplementary material for: A SNP variation in an expansin (EgExp4) gene affects height in oil palm
Source: PeerJ. 2022 Mar 16;10:e13046. doi: 10.7717/peerj.13046 (PMC8934041; doi:10.7717/peerj.13046)
Supplement: Supplemental Information 6 [file peerj-10-13046-s006.pdf]

***EgGA20ox1* Full length Information**

Length: 2759 bp (Same as report)
START Codon: 886-888 (ATG GCC ACC) (Checked)
STOP Codon: 2221-2223 (TGA TGA CAC) (Checked; Different from report)
G → GAA insertion : 1428
G → GA, GAAA insertion : 1428
T → G SNP : 1468

BLAST Information
Name: gibberellin 20 oxidase 1-D-like [*Elaeis guineensis*]
Sequence ID: XP_010934079.1
Exon 1: 697-1394
Exon 2: 1517-1839
Exon 3: 1946-2474

Legend
= Primer
= START/STOP codon
= G → GAA insertion. Insertion found in some short oil palm
= G → GA, GAAA insertion. Insertion found is some tall oil palm
= T → G SNP. Base G found in some short oil palm

```
1     TGTTgGCTTG AGCATaGGAC TCATATCTAT TTCtGCCAAT CAAAGATTGA

51    TATAAATATA GATAGATAAA TAAATGACTA TATTAATATG ATATTAACTA

101   TTTgTTGTAA TTTGCTTTcC TTTTATTgGC AAGATaCGGC CGCAAGaAtA

151   gGTTTACTTT TTGGTTGCCA ATAGCAAACT CCTGTATTCA GAAGAAATTA

201   TTAGATGGAT TAGGTTTAGT TGGCCAATTC AAATCCCAGA GCACATGCAC

251   GTTTTTACTA TACATGTGCC CTAAAATTTA GATTGGTCCA TTGGATCTAG

301   TCTATCTAAC AATCTGGCTA CATTGAACCC tCACCTCAAA GAATGGAGAA

351   ACTACTAAAC GGACCACATC GGATCAGTCC AAATCCCGAA ACACGCGTAC

401   AGTGCATGAT AGCATGAATT CTCTCCTCTG TTTTGATGTT GAATTATTTA

451   AATAAAATGG GGTAACGTGG AAAGTGACAA TTGGATGGAC TTATTGGACG

501   TGATCCATCT ACTAACTTGA ACCTTACATA TACTCATTGT CATACTCCTG

551   AAGTATTAAA ATGATATCAT TTATaAAAAA ATATTCACGA TGGTTCTGAT

601   TTGCTGCACC CAGAATTACC CACTGTGGAG TCAAACCACA AGCCTTGTTC
```

[cont. *EgGA20ox1* Full Length Sequence]

651 CTCCCACTAT AAAATGGGCA GCAGCCAGGC CAAATGATAC GAAGAGATTG

701 AATCCTTTAA GATTCCATAT TTTTCTATAC CGGCTTCTCT CCTCCCACTC

751 CCACTCCCAC TCCCActcCT CCCATTCTGC AACCCCATCC CTTCTTTCCT

801 TCCTCTCCtC TTTCTCCATC CACCCCTGGT ACTCCGCTCT CTCGcCCCAC

851 CGGAGGCCGA ACAGGAGGCg CGGGCCCCGC AGCCCATGGC CACCGCCGCC

901 CAGCAACCGC CGTCGCTGGt CTTTGACGCC GCCGtCCTCA GCCAGCGGCC

951 GGACATTCCG GTCGAGTTCG TCTGGCCGGA GGAGGACAAG CCCACCCCGG

1001 ACGCCGCCGA GGAGCTGTCC GTCCCCCTGA TCGACCTCGG CGACTTCCTC

1051 TCCGGCGACC CCGCCGCCGT GGCCGACGTT TCCCGACTCG TCGGCGAGGC

1101 GTGCAGTCGG CACGGCTTCT TCCACGTCGT CAACCACGGC ATCCCCCTCGG

1151 CGCTCTTCGC CAAGGcCCAC CGCTGCGTCG ATGGCTTCTT CTCGATGCCG

1201 CTCGCCGAGA AGCAGAGGGC CCAGCGAAAG CCCGGGGAGA GCTGCGGCTA

1251 CGCTAGCAGC TTTACCGGGA GATTCGCTAA TCGGCTCCCA TGGAAGGAGA

1301 CCCTTTCTTT TCGCTTCTCC TCCTCTCCTC TCTCCCCAAA TATCGTGCAG

1351 GACTACTTCG TCCGCACTCT TGGCGAAGAT TTCCTGCACT TTGGGTAGGA

1401 CAGAATAACA AAACaAGACT GCGACATGaa aaaAAAAAAA ACTaCTAATA

1451 ATaGaaaAAA AAAcaAtTGt ttTTTTTTTT cCtTATAAAT TTCCTCTCAT

1501 TACTGTGGTT CTACAGCACG GTGTACCAAG ACTACTGCAA TGCGATGAGC

1551 AAGCTGTCAT TGGCGATAAT GGAGATTATC GGGACGAGTC TGGGGGTGGG

1601 GCGGGCATAT TATCGGGAGT TCTTCGAAGG GAATGATTCG ATAATGAGGC

1651 TGAACTACTA CCCACCGTGC CAGAAGCCGG ACCTGGCCCT CGGCACCGGT

1701 CCCCATTGCG ACCCCACCTC CCTGACCATC CTCCACCAGG ACGACGTCGG

1751 CGGCCTTCAG GTGTTCACCG ACGGCAAGTG GCGCACTGTC AGCCCCAAAC

1801 CGGATGCCCT TGTTGTCAAC ATCGGTGACA CCTTCATGGT ACACAATATC

1851 TATACAATCA ACTCTTGGAC TTGGAATTAA TATTTCGAGA TCTTCGATCG

[cont. *EgGA20ox1* Full Length Sequence]

1901 TATTATGAGA AGGGATGGTC TTCGTTGTAA TATTGGATTT GCAGGCGTTG

1951 TCGAATGGGA GGTACAAGAG CTGTCTGCAC CGGGCGGTGG TGAACAGCAA

2001 GGTGGCGAGG AAGTCTTTTG CCTTCTTTCT GAGCCCGGAG ATGAACAAAA

2051 TAGTGCGGCC GCCGGTGGGG CTGGTGGACG CGGAGCACCC GAGGGCCTAC

2101 CCGGACTTCA CGTGGTCGGC GCTGCTCGAG TTCACCCAGA AGCACTACCG

2151 GGCCGATATG AACACCCTCG ACGCCTTCAC CAAGTGGATG ATGCTCCAGG

2201 CCGAGGGGCC CGTGCCGCAG TGATGACACG GCTGGGGGAT TTTTCTATCA

2251 ATTTTTTAAG TCATTAAACC TCTTGGATTT AACTAGCTAG CTAGCTTATA

2301 GGGGGTCGGA TACAGAGGAA GGTTATGGTG TGAAGGAATA ATGAGGCTAG

2351 GGAATGAAAT AAAAGTAAAA GGAGGaagTG AAACGTACGC TTGTTTCCAA

2401 CTCTCATCGG AGCCAGCCAC TGTTAATACC ACTGTGAAGT AAATTGCCCG

2451 TAAAGGCCAG CACATCTCTT GTCATTACAC TCTTTCTCTC TCTTTTTCAT

2501 CCTTTATTTC ATATTTGAAC AAAACTTATA ACACGGGGAG AAACAGGGGT

2551 GGGAGAATCC ACGCCTTTAG GCTATGATAG GAGGGGCAGG CAAAAGAACA

2601 AGCTTCGGTG GCACATGTTT TTAGGAGAAC AGGAGATGGT GATGATGACG

2651 GAGGGACGAC GACATACCAA AGAGGCGGAC GGCTCGATAT CTCCGCAGCA

2701 CTGGCTTGTC CTCTCCTGCG ACTGGATTTT TTCTATTTCT CTTTTGTCAC

2751 CTAGCGCCT

\\end of sequence

Fig. S6 The insertion and deletion variations are illustrated on the *EgGA20ox1* reference gene sequence, which has a full-length of 2759 bp (oil palm draft sequences of Malaysian Palm Oil Board (MPOB), (http://genomsawit.mpob.gov.my/genomsawit/)).
