# Supplementary material for: A SNP variation in an expansin (EgExp4) gene affects height in oil palm
Source: PeerJ. 2022 Mar 16;10:e13046. doi: 10.7717/peerj.13046 (PMC8934041; doi:10.7717/peerj.13046)
Supplement: Supplemental Information 7 [file peerj-10-13046-s007.pdf]

***EgExp4* Full length Information**

Length: 2586 bp (Same as report)
START Codon: 1045-1047 (Checked)
STOP Codon: 2006-2008 (Checked)
T → C SNP: 118
TC → T deletion: 989

BLAST Information
Name: expansin-A2 [*Elaeis guineensis*]
Sequence ID: XP_010938009.1
Exon1: 1005-1119
Exon2: 1304-1616
Exon3: 1706-2386

Legend
= Primer
= START/STOP codon
= T → C SNP. Base C found in some tall oil palm
= TC → T deletion. Deletion found in some tall oil palm
Underline = Exon

EgExp4 Full Length Sequence

```
1     TGAGGGCTAC AATTTTGAAT GATTGGTATC CCTGTGTAGG TGAATATTCA

51    ATACTAGAGG CAGTAATAAC AACATACGTA ATATGATAAT TCATGAAAGA

101   TACGATAAAA TTATTGGTGA ATAATTTGTT aAAATAAATC CTTACATCAT

151   TCAAATGATA TTAGGTGATT CAATTATCTA AGAGTAATCT AGAGCATCAT

201   CGAATCAACA CaAAAAAATA TGGCTAACTA GAGAATGCTT CAACAATTCT

251   GAGAACACAA TCATTGCTTA GATTGATAGG CTGATAGAAT ATATATCTTG

301   CTTGAAGGAA AAGATAAGAC CATTAAATTA AGTTTGAGAA CACGGATACT

351   AATCATTAGA TATAACCAAC TAACCTGCCT CATTCCTCAT TACCTAGCAT

401   TCTATGCAAA ATATTGCACT TAAGCCATTA TTTAGGATCT TTGAAAATAT

451   AACATATATA TATAGCTCAT TAATTGCAAT TTTACTTTTc CCCCCAAAAT

501   TAAACACAAT TGCATGCTTC TTCAATGTAA TCTTTACCAA CCATTTGGTA

551   CTTTCATAGT GCACTTAATT GTGCAGTTGT ATAAAGTTCC CTTGATAGCT

601   TAAGTTATAT TAATAATATT ATTATTTGGA ACATGGACAT ATCCACGCCT

651   CCTCTATATC CACATCTATA CTTGGATAGA ATAATTCATA TATATCAATA
```

[cont. *EgExp4* Full Length Sequence]

701 TCACATTTAT ATAGCGGTGC TCTCAGAGAA AAAGAAAGAA AGAAATaAAA

751 ATATTTTCGT AGCGGTGGAA AATTGGACCG GGACACGGAG AGAAGATTCC

801 TTGGGAGGAG GTGCATGTGC GTGAGGACCC CAATTGGGCT CAGCTGGTAC

851 TTCACCCAGA ACATTTGCAT GTGCGCTTGA TTTCCATAAT CGCTGCAATC

901 CGTTCCAAGC CTTTGCCCGG GGCCAGCGTG ACAACAACCT CCTTCGTCTC

951 TTTATACTCC TCTCCCCTAT ATATAGCCCT CACCTCCCTC CCCCCCTTCG

1001 CCAACCAAGC AACAAACACC TCCATTCCTT CGTACTCCTC TCCAATGGCG

1051 TCCCCAAAGT TCTCGCTCTC GAACTCCGCC CTCTCCTTCT TCTTCTTTGC

1101 TTCCTTGTGC TTCTTTGCAT CGCAAGCCTT GGGGGACTCC GGATGGCAGA

1151 ACGCACACGC CACGTTCTAC GGCGGTGGCG ATGCCTCCGG CACGATGGGT

1201 GAGTCGATTT GTTCTCCGGG ATAACAAGAC TTGGCTCGCT GTTTAATTTA

1251 GCTGCTTGCC AACTTCTCCT CATTGGTGCA AATTGGtTTT TTTTGGTTGC

1301 AGGAGGGGCT TGTGGATATG GCAACCTCTA CAGCCAGGGG TACGGGACCA

1351 ACACCGCGGC CCTCAGCACC GCTCTCTTCA ACAACGGGCT CAGCTGCGGC

1401 TCGTGCTACG AGATGCGGTG CGCTGACGAC CCCCAGTGGT GCCTCCCAGG

1451 CTCCATCATC GTCACCGCCA CCAATTACTG TCCCCCGAAC TACGCCCTCC

1501 CCAACGACAA CGGCGGGTGG TGCAACCCTc CCCGCGAGCA CTTCGACCTC

1551 GCCGAGCCTG CCTTCCTCCA AATTGCCCAG TACCGTGCCG GCATCGTCCC

1601 CATCTCCTAC CGTAGGTGAG CATTGTCTAT ATAACCAATC TCTCAAGTCA

1651 TATCTCAAAA GTGATCGACT TTATGAACTG TACTACGTGA TAAATATCTG

1701 GTAGGGTCCC CTGTGTGAAG AAGGGAgGGG TAAGGTTCAC CATCAATGGC

1751 CACTCCTACT TCAACTTGGT TCTCATCACC AATGTCGCTG GGGCCGGAGA

1801 CGTGCATGCG GTGTCGATCA AGGGGTCCAA CACCGGATGG CAGGCCATGT

1851 CGCGAAACTG GGGCCAGAAT TGGCAGAGCA ATGGCTACCT CAACGGCCAG

1901 AGCCTCTCCT TCCAGGTCAC CTCCAGCGAC GGGAAGACCG TCACCAGCTA

[cont. *EgExp4* Full Length Sequence]

1951 CAACGTCGTG CCGGCCAACT GGCAGTTCGG CCAGACCTTT GAGGGAGAAC

2001 AATTCTAGGC CTCCATTGTT AgGGGGGTCT TAAGGGTTTG GGGTTTGGGA

2051 GGATGGCCTC TCTTTGACTT TGAGAGAGGT TTGGGCCTGG TTGAATGCTG

2101 AGGTGGCTTT ATTAGCACCC GCTGAGATGG CCTAAATAGT AGACATATTA

2151 GACATAGATG GTCGTCTTTG TTTCTTGTTG GCAATTTAGA TTGAATGCCT

2201 TGTGGAGATT AAGAAATCAA GGCCTTGGGT GTATAGAATT GGAATCCTCA

2251 ACATATTATA ATATGATTGT GTGGGGGATG GTATGTCAAT TAGAGGAAAG

2301 TATGATGTTT GCTTTGTGTA TTTCAAACAT AATATTTGTA ATCGATTGTT

2351 CTCCAATAAT AATAAGCAAT TTATTAATTG CAGCAATGGA TTGTTCCGTT

2401 ATTATTATAT TAtTTTTTTC TAAGGATCTT CTGACATGGA CCAGAAGCCA

2451 TGTTTGAGGA TTCTATTTGG CCTCAGGAAA AAGATGTGGT CCTTAACCTG

2501 ACCTGTTTGC ACAACAGGTT TGTTCAAGTT TGCAACGTGT GATGGATGCT

2551 CGGCATCACA GGACTAACCA ATCCTGGCAA GATGAC

\\end of sequence

Fig. S7 The insertion and deletion variations are illustrated on the *EgExp4* reference gene sequence, which has a full-length of 2586 bp (oil palm draft sequences of Malaysian Palm Oil Board (MPOB), (http://genomsawit.mpob.gov.my/genomsawit/)).
