# Supplementary material for: A SNP variation in an expansin (EgExp4) gene affects height in oil palm
Source: PeerJ. 2022 Mar 16;10:e13046. doi: 10.7717/peerj.13046 (PMC8934041; doi:10.7717/peerj.13046)
Supplement: Supplemental Information 8 [file peerj-10-13046-s008.pdf]

***EgAPG1* Full length Information**

Length: 2917 bp (Same as report)
START Codon: 1059-1061 (Checked)
STOP Codon: 2800-2802 (Checked)
No variation found between short and tall oil palm

BLAST Information
Name: stem-specific protein TSJT1-like [*Elaeis guineensis*]
Sequence ID: NP_001306846.1
Exon 1:   Identities 191/260 (73% Match)
Exon 2:   Identities 65/65 (100% Match)

Legend
= Primer
= START/STOP codon

EgAPG1 Full Length Sequence

```
1     tGGCACCTCG ATGTATTTGA GGTCaAAAAA AAGaTAATAC CTCATCAAAA

51    CAAATATAGA GATTGATCCA TCCGATCTAT GATCCAAATC ATCCGAAcCC

101   CGATCACAAT AAGAATcCAT GCAAAcGTTG TATTTAATCA CTCGTTCAtC

151   aTATAGATAT TGAATATTTG CACTaAAAAA AGTAGAACAA AAtTTTTcAT

201   TAATCTAGAT AAaATTTTAG ATTATTATAA TTTATAATGG CAACAGaTGA

251   TTATGAAGTA TCGAAGGGTG TTGCAGCTAg GaTTTTAGTA TCCAAGATAC

301   TATTAGCCTT CTCTATGTTT GTCTGTTTTG TGTTTCTTTC TCCATCTTGT

351   AAAAATaAAA TAAAGGGGCG ATTATGGAGA CAGTCCCGCA GACAGGGCAA

401   GGAATCCAAG AGTGCGATTT ACCGTCGTCT CCGGAAACGG AGGAGGTTGA

451   CGGGGTCTTC TCGGGACGTT AGGAGAAGGA TCGGACCACA CATCGTCCGC

501   CAAAAACAAC CCCCGAGTGG GACCCACAGT GGTCCTCTTT TTCCTTTGTT

551   TCGTTTTTCT AATTTAATTT TTGCCCTCTC AAGCTAAAAA TATTCCCATC

601   CGCGGGCTGG CAGATGTCGA CGTGGCACCT CGTACACAGA GATATTTTCT

651   TAAACCAGTG GGCGGTAGCG TGGATCCGCG TCAGCGGATA AGTTCTCCAC

701   AGcGGCCTCC CCTTCGTACC ACTGCAGAAA CACCGGATCT GGCCCGATTT

751   ACGGCCCCCA CCCACGAGAC CAAGTCAATT GTGTGACGTA AGGCGTGAAC
```

[cont. *EgAPG1* Full Length Sequence]

801 GGTAACCACA CCCGGAAGTG TTCATATCTC GCATGTCTGG AATAGCTGCG

851 CAATTCTAGC ATGGGGAATA GGGATCCTTA TCCTCCCATC CCTATCCACT

901 TCCACCTCCC AATTGAAAAA ATGTACTACt ATACCACCGG ACACAACTAT

951 ATATACAcCT TCCCTaTCTC TTGCCAAAAC TCCATTCCCC TCCGTTCCCT

1001 TTCCTTCTCC TATTTGCTTT ACTTTTCTTG AGTTTTAGAC GCAGAGAGAA

1051 TAGAGAAGAT GTTGGCGATA TTCCACAAGG CGTTCGCTCA CCCGCCCCAG

1101 GAGCTCAACA GCCCCGGTGG CGGCCGGCGT GTCCCCAAGA ACCCGGAGGA

1151 GATCCTCCGG GAGTTCCACT CCCTCCACCC TGGGGACTCC TTCTCCGCCA

1201 CCTTCAGCGG CGGCGCCGCC CTCGCCTGCG TCCCCTCCCA CTCCAACCAT

1251 TCTCCTCAGC AAAGGCACAG CTTTTTTAtT CCCATATATT CCAGCTTCCA

1301 ATTAAGCATC GCAAATATAT GACACCCTCT GTTTTTATTT GGGGGCAGGT

1351 TGTTCTGTAG CTTTGATGAC ATATATTGTA TGTTCGTGGG GGGACTCGAC

1401 AACTTGTGCT CTCTTATTAG GCAGTACGGG CTAAGCAAGA ACACGAACGA

1451 GGCTTTGCTT GTGATCGAGG CGTACCGGAC GCTGCGCGAC CGCGGCCCGT

1501 ACCCGGCGGA CCAGGTCCTC AAGGACCTCG GCGGCTCCTT CGGCTTCGTG

1551 CTCTACGATA GCAAGGCCGG CACCGTCTTC GCTGCGCTGG TATAAATTTA

1601 TTCTCGGTTT ATTCCGTTTC TTCTTCTTCT TCTTCTTCTT CTTCGTGGCT

1651 GCTGTGGCTG ATGGGGAGTT GTTGTTCTTG tTTTTTGGAA TTgGGGTgGG

1701 GTGCAGAGCG CGGATGGaAA GATCCCGCTG TTCTGGGGCA TCGCCGCGGA

1751 TGGATCGGTT GTGATCTGTG ATGATGTGGG GATTATAAAA GGAGGCTGCG

1801 GCAAATCATA CGCTCCTTTc CCAACTGGTA TGtTTTTTTT TCtAAATctT

1851 aTTATTATTA TTATTATTTA AGTTTTAGTA TATGAATATG CTGTAATTAG

1901 TTTAAGATTT ATATGCAAAT CtTaTTATTA TTATTATTAT TATTATGGtT

1951 TTTTAAGTTT TAGTATATGA ATATGCTGTA ATTAGTTTAA GATTTATATG

2001 CAAATCTGTT TTAATTGTAA GAGTATGGAG GGAGGGAAGG AGGAAATTAA

[cont. *EgAPG1* Full Length Sequence]

2051 GGCCAAATTA AccttGATTT TGTTACCATA AATATGGCAT ATATAATTAA

2101 TTACTTGAtG GAGAACCATg cCCTGTTTGG TTCCAGCATA CTTTTgGTCG

2151 GTTAATTTGA ATAGGAGGAA TAAGTTCGGG GCTAGAACAG AGGGATAAAG

2201 CTGACAAAGT CTGGATTAAA TTATTCAACT ATCTTAATCG aAAAAAAATA

2251 TTCCGATTAA AGAGGATAAG GTACTaaAAA AAAATTAATC TCAAAAGTAT

2301 GATCCATTAT CTTCATTCCA AAAAAGATGC AGAATTAAAC TGAATAAGTC

2351 GGTATTTAAT TATTAATCAA TCACCGGCCA GagGAGTCCC ACATGTTAGA

2401 TCTCgGATCA AATCTAAAAC AGAGCCTTCA GTTGAAGCGA ATTGGCATAA

2451 CATAATTATT GGTAATATGA CATGGATGTG ATTCATGTGA TCGAGTGGTA

2501 ACACCCCAAG GGAGATTTGG CAAACTATTA TAATTTCCAT GAATTTGTTC

2551 TTGAGCTCAA ATTACAACAT CAATGTTATA TATTTAATGA CAATATTGGA

2601 TACAGGGTGC ATGTTCCATA GCGAGGGAGG ACTGAAGAGC TTCGAGCATC

2651 CCATGAACAA GCTGAGGCCA ATGCCGAGGG TGGATAGCGA GGGGGTGATG

2701 TGCGGTGCtA ACTTCAAGGT CGACACCTAT TCCAGGATTA ACAGCATGCC

2751 ACGAGTCGGC AGCGCCACCA ACTGGACCTT CTGGGACGAA TCCATGTTGT

2801 AGCTTAGCTT TCTACTCTTG GCAACAACAA CAACAACAAG AAACCAATCC

2851 TATCTTAAGT CTGCCTTTCT TCTGCCTCAT CTTCTTTGTT TAAATGGGTT

2901 TCGAGTTCCC TTTGCAG

\\end of sequence

Fig. S8 The insertion and deletion variations are illustrated on the *EgAPG1* reference gene sequence, which has a full-length of 2917 bp (oil palm draft sequences of Malaysian Palm Oil Board (MPOB), (http://genomsawit.mpob.gov.my/genomsawit/)).
