# Supplementary material for: A SNP variation in an expansin (EgExp4) gene affects height in oil palm
Source: PeerJ. 2022 Mar 16;10:e13046. doi: 10.7717/peerj.13046 (PMC8934041; doi:10.7717/peerj.13046)
Supplement: Supplemental Information 9 — The specified primers were selected based on their size amplification in the expected size. The amplification sites were from 5′ UTR to 3′ UTR sites of the genes. [file peerj-10-13046-s009.docx]

**Table S1** Details of gene-specific primers designed from the height-related genes, including *EgDELLA1*, *EgGRF1*, *EgGA20ox1*, *EgAPG1* and *EgExp4*. The specified primers were selected based on their size amplification in the expected size. The amplification sites were from 5՛ UTR to 3՛ UTR sites of the genes.

| Order | Gene name | Chr. | Gene sequence (bp) | Primer name | Primer sequence (5'-3') | size (bp) | Selected primer |
| --- | --- | --- | --- | --- | --- | --- | --- |
| 1 | *EgDELLA1* | 14 | 1739 | **EgDELLA1-P1F** | **TTTTCGTACATTCGGCTCTG** | **3015** | **✓** |
|  |  |  |  | **EgDELLA1-P1R** | **ATCCTGTTCGAAAGCGAGAA** |  |  |
|  |  |  |  | EgDELLA1-P2F | GGCATAGCTTCTCTCTCCTATGC | 3384 |  |
|  |  |  |  | EgDELLA1-P2R | GTCAAGAACCAGGAGCAAGC |  |  |
| 2 | *EgGRF1* | 10 | 1038 | EgGRF1-P1F | CAGGAGACAACAGCAGCAAA | 2635 |  |
|  |  |  |  | EgGRF1-P1R | CAATGACATGCAACATTCCTG |  |  |
|  |  |  |  | **EgGRF1-P2F** | **TCATTGTTCCACACTTCCACA** | **2516** | **✓** |
|  |  |  |  | **EgGRF1-P2R** | **GGAGAAGATCAGAAGACTGGTTACA** |  |  |
| 3 | *EgGA20ox1* | 11 | 1341 | EgGA20ox1-P1F | TGAGAGACAAAAACGAACAGGA | 2538 |  |
|  |  |  |  | EgGA20ox1-P1R | CAAGAGATGTGCTGGCCTTT |  |  |
|  |  |  |  | **EgGA20ox1-P2F** | **TGTTGGCTTGAGCATAGGACT** | **2759** | **✓** |
|  |  |  |  | **EgGA20ox1-P2R** | **AGGCGCTAGGTGACAAAAGA** |  |  |
| 4 | *EgAPG1* | 16 | 1744 | **EgAPG1-P1F** | **TGGCACCTCGATGTATTTGA** | **2917** | **✓** |
|  |  |  |  | **EgAPG1-P1R** | **CTGCAAAGGGAACTCGAAAC** |  |  |
|  |  |  |  | EgAPG1-P2F | TCCACATCGATTGTGAGTTAAGA | 3332 |  |
|  |  |  |  | EgAPG1-P2R | GCACCACCTTAGGCATTGAT |  |  |
| 5 | *EgExp4* | 14 | 964 | **EgExpa4-P1F** | **TGAGGGCTACAATTTTGAATGA** | **2586** | **✓** |
|  |  |  |  | **EgExpa4-P1R** | **GTCATCTTGCCAGGATTGGT** |  |  |
|  |  |  |  | EgExpa4-P2F | TTGGTATCCCTGTGTAGGTGAA | 3168 |  |
|  |  |  |  | EgExpa4-P2R | AAAATGGAGAAGGTTCGGTTT |  |  |
