# Supplementary material for: A SNP variation in an expansin (EgExp4) gene affects height in oil palm
Source: PeerJ. 2022 Mar 16;10:e13046. doi: 10.7717/peerj.13046 (PMC8934041; doi:10.7717/peerj.13046)
Supplement: Supplemental Information 10 — Sequences in red letters represent forward M13 sequence (18 bp) while sequences in blue letters represent reverse M13 sequence (18 bp). [file peerj-10-13046-s010.docx]

**Table S2** Details of M13-tagged primers for full-length genomic DNA amplification, including M13-tagged EgDELLA1-P1, EgGRF1-P2, EgGA20ox1-P2, EgAPG1-P1 and EgExp4-P1 primers. Sequences in red letters represent forward M13 sequence (18 bp) while sequences in blue letters represent reverse M13 sequence (18 bp)

| Primer name | 5' to 3' primer sequences |
| --- | --- |
| EgDELLA1-P1F_M13F | /5AmMC6/TGTAAAACGACGGCCAGTTTTTCGTACATTCGGCTCTG |
| EgDELLA1-P1R_M13R | /5AmMC6/GGAAACAGCTATGACCATGATCCTGTTCGAAAGCGAGAA |
|  |  |
| EgGRF1-P2F_M13F | /5AmMC6/TGTAAAACGACGGCCAGTTCATTGTTCCACACTTCCACA |
| EgGRF1-P2R_M13R | /5AmMC6/GGAAACAGCTATGACCATGGGAGAAGATCAGAAGACTGGTTACA |
|  |  |
| EgGA20-P2F_M13F | /5AmMC6/TGTAAAACGACGGCCAGTTGTTGGCTTGAGCATAGGACT |
| EgGA20-P2R_M13R | /5AmMC6/GGAAACAGCTATGACCATGAGGCGCTAGGTGACAAAAGA |
|  |  |
| EgAPG1-P1F_M13F | /5AmMC6/TGTAAAACGACGGCCAGTTGGCACCTCGATGTATTTGA |
| EgAPG1-P1R_M13R | /5AmMC6/GGAAACAGCTATGACCATGCTGCAAAGGGAACTCGAAAC |
|  |  |
| EgExpa4-P1F_M13F | /5AmMC6/TGTAAAACGACGGCCAGTTGAGGGCTACAATTTTGAATGA |
| EgExpa4-P1R_M13R | /5AmMC6/GGAAACAGCTATGACCATGGTCATCTTGCCAGGATTGGT |
