# Supplementary material for: A SNP variation in an expansin (EgExp4) gene affects height in oil palm
Source: PeerJ. 2022 Mar 16;10:e13046. doi: 10.7717/peerj.13046 (PMC8934041; doi:10.7717/peerj.13046)
Supplement: Supplemental Information 11 [file peerj-10-13046-s011.docx]

**Table S3** Details of barcode (BC)-tagged M13 primer sets for both BC forward and BC reverse primers.

| **BC forward primer** | **primer sequence (5՛-3՛)** | **BC reverse primer** | **primer sequence (5՛-3՛)** |
| --- | --- | --- | --- |
| F-M13BC-01 | TCAGACGATGCGTCATTGTAAAACGACGGCCAGTT | R-M13BC-01 | TCAGACGATGCGTCATGGAAACAGCTATGACCATG |
| F-M13BC-09 | CTGCGTGCTCTACGACTGTAAAACGACGGCCAGTT | R-M13BC-09 | CTGCGTGCTCTACGACGGAAACAGCTATGACCATG |
| F-M13BC-10 | GCGCGATACGATGACTTGTAAAACGACGGCCAGTT | R-M13BC-10 | GCGCGATACGATGACTGGAAACAGCTATGACCATG |
| F-M13BC-17 | CATAGCGACTATCGTGTGTAAAACGACGGCCAGTT | R-M13BC-17 | CATAGCGACTATCGTGGGAAACAGCTATGACCATG |
| F-M13BC-19 | CGCATCTGTGCATGCATGTAAAACGACGGCCAGTT | R-M13BC-19 | CGCATCTGTGCATGCAGGAAACAGCTATGACCATG |
| F-M13BC-21 | GTACACGCTGTGACTATGTAAAACGACGGCCAGTT | R-M13BC-21 | GTACACGCTGTGACTAGGAAACAGCTATGACCATG |
| F-M13BC-29 | GCTCGACTGTGAGAGATGTAAAACGACGGCCAGTT | R-M13BC-29 | GCTCGACTGTGAGAGAGGAAACAGCTATGACCATG |
| F-M13BC-34 | TGCTCGCAGTATCACATGTAAAACGACGGCCAGTT | R-M13BC-34 | ACTCTCGCTCTGTAGAGGAAACAGCTATGACCATG |
| F-M13BC-38 | ACTCTCGCTCTGTAGATGTAAAACGACGGCCAGTT | R-M13BC-38 | TGCTCGCAGTATCACAGGAAACAGCTATGACCATG |
| F-M13BC-40 | CAGTGAGAGCGCGATATGTAAAACGACGGCCAGTT | R-M13BC-40 | CAGTGAGAGCGCGATAGGAAACAGCTATGACCATG |
| F-M13BC-48 | TCACACTCTAGAGCGATGTAAAACGACGGCCAGTT | R-M13BC-48 | TCACACTCTAGAGCGAGGAAACAGCTATGACCATG |
| F-M13BC-52 | GCAGACTCTCACACGCTGTAAAACGACGGCCAGTT | R-M13BC-52 | GCAGACTCTCACACGCGGAAACAGCTATGACCATG |
| F-M13BC-54 | GCAGACTCTCACACGCTGTAAAACGACGGCCAGTT | R-M13BC-54 | GTGTGAGATATATATCGGAAACAGCTATGACCATG |
| F-M13BC-62 | CTGCGCAGTACGTGCATGTAAAACGACGGCCAGTT | R-M13BC-62 | GACAGCATCTGCGCTCGGAAACAGCTATGACCATG |
| F-M13BC-70 | GACAGCATCTGCGCTCTGTAAAACGACGGCCAGTT | R-M13BC-70 | CTGCGCAGTACGTGCAGGAAACAGCTATGACCATG |
