# Supplementary material for: A SNP variation in an expansin (EgExp4) gene affects height in oil palm
Source: PeerJ. 2022 Mar 16;10:e13046. doi: 10.7717/peerj.13046 (PMC8934041; doi:10.7717/peerj.13046)
Supplement: Supplemental Information 12 — To amplify each SNP position, three primers, including two forward primers that are specific for each SNP (red letters and yellow highlights) and 1 common reverse primer, were designed. Sequences in blue letters represent sequences for FAM emission while sequences in green letters represent sequences for HEX emission. [file peerj-10-13046-s012.docx]

**Table S4** Details of SNP primers designed from the 4 SNP variations of the three genes, including *EgDELLA1*, *EgGA20ox1* and *EgExp4*. To amplify each SNP position, three primers, including two forward primers that are specific for each SNP (red letters and yellow highlights) and 1 common reverse primer, were designed. Sequences in blue letters represent sequences for FAM emission while sequences in green letters represent sequences for HEX emission

| SNP primer name | Primer Sequences | SNP1 | SNP2 |
| --- | --- | --- | --- |
| mEgDELLA1_SNP2100_Allele 1 | GAAGGTGACCAAGTTCATGCTCCTCCTCCAATTCAAACATCTCTTCA | T | A |
| mEgDELLA1_SNP2100_Allele 2 | GAAGGTCGGAGTCAACGGATTCCTCCTCCAATTCAAACATCTCTTCT |  |  |
| mEgDELLA1_SNP2100_common reverse primer | AAGCAGTGGCAGTCTCAGATCTCAA |  |  |
|  |  |  |  |
| mEgDELLA1_SNP2248_Allele 1 | GAAGGTGACCAAGTTCATGCTGCCATACGTAATGATAGTTGCAGATG | G | A |
| mEgDELLA1_SNP2248_Allele 2 | GAAGGTCGGAGTCAACGGATTAGCCATACGTAATGATAGTTGCAGATA |  |  |
| mEgDELLA1_SNP2248_common reverse primer | GGCTTCAGTGAAGCGGTTGATGAAA |  |  |
|  |  |  |  |
| mEgGA20ox1_SNP1468_Allele 1 | GAAGGTGACCAAGTTCATGCTATGAGAGGAAATTTATAAGGAAAAAAAAAAACA | T | G |
| mEgGA20ox1_SNP1468_Allele 2 | GAAGGTCGGAGTCAACGGATTATGAGAGGAAATTTATAAGGAAAAAAAAAAACC |  |  |
| mEgGA20ox1_SNP1468_common reverse primer | TGCACTTTGGGTAGGACAGAATAACAAAA |  |  |
|  |  |  |  |
| mEgExp4_SNP118_Allele 1 | GAAGGTGACCAAGTTCATGCTATAATTCATGAAAGATACGATAAAATTATTGGT | T | C |
| mEgExp4_SNP118_Allele 2 | GAAGGTCGGAGTCAACGGATTATAATTCATGAAAGATACGATAAAATTATTGGC |  |  |
| mEgExp4_SNP118_common reverse primer | CTCTTAGATAATTGAATCACCTAATATCAT |  |  |
|  |  |  |  |
