# Supplementary material for: A SNP variation in an expansin (EgExp4) gene affects height in oil palm
Source: PeerJ. 2022 Mar 16;10:e13046. doi: 10.7717/peerj.13046 (PMC8934041; doi:10.7717/peerj.13046)
Supplement: Supplemental Information 13 — Genotype C/C was shown to be tallest, at 210 cm for HT-1, 250 cm for HT-2, 278 cm for HT-3 and 409 cm for HT-4. Genotype T/T was shown to be shortest, 185 cm for HT-1, 224 cm for HT-2, 257 cm for HT-3 and 370 cm for HT-4. [file peerj-10-13046-s013.docx]

**Table S5** Height details of three mEgExp4-SNP118 genotypes, including C/C, T/C and T/T, for height-recordings HT-1, HT-2, HT-3 and HT-4 from the GT population. Genotype C/C was shown to be tallest, at 210 cm for HT-1, 250 cm for HT-2, 278 cm for HT-3 and 409 cm for HT-4. Genotype T/T was shown to be shortest, 185 cm for HT-1, 224 cm for HT-2, 257 cm for HT-3 and 370 cm for HT-4.

| Trait | Loci | Number | Mean (cm.) | Std. Deviation (cm.) |
| --- | --- | --- | --- | --- |
| HT-1 | CC | 25 | 210 | 38 |
|  | TC | 78 | 193 | 32 |
|  | TT | 66 | 185 | 35 |
|  | Total | 169 | 192 | 35 |
| HT-2 | CC | 25 | 250 | 44 |
|  | TC | 78 | 232 | 37 |
|  | TT | 66 | 224 | 40 |
|  | Total | 169 | 232 | 40 |
| HT-3 | CC | 25 | 278 | 45 |
|  | TC | 78 | 263 | 42 |
|  | TT | 66 | 257 | 46 |
|  | Total | 169 | 263 | 44 |
| HT-4 | CC | 25 | 409 | 53 |
|  | TC | 78 | 380 | 57 |
|  | TT | 66 | 370 | 61 |
|  | Total | 169 | 380 | 59 |
